# Chaperone Copolymer-Assisted Catalytic Hairpin Assembly for Highly Sensitive Detection of Adenosine

**DOI:** 10.3390/polym16152179

**Published:** 2024-07-31

**Authors:** Yazhen Liao, Xiaoxue Yin, Wenqian Liu, Zhenrui Du, Jie Du

**Affiliations:** School of Materials Science and Engineering, Hainan University, Haikou 570228, China; 21220856000036@hainanu.edu.cn (Y.L.); 22220856010035@hainanu.edu.cn (X.Y.); 20213001771@hainanu.edu.cn (W.L.); 20223000742@hainanu.edu.cn (Z.D.)

**Keywords:** chaperone copolymer, catalytic hairpin assembly, adenosine

## Abstract

Adenosine is an endogenous molecule that plays a vital role in biological processes. Research indicates that abnormal adenosine levels are associated with a range of diseases. The development of sensors capable of detecting adenosine is pivotal for early diagnosis of disease. For example, elevated adenosine levels are closely associated with the onset and progression of cancer. In this study, we designed a novel DNA biosensor utilizing chaperone copolymer-assisted catalytic hairpin assembly for highly sensitive detection of adenosine. The functional probe comprises streptavidin magnetic beads, an aptamer, and a catalytic chain. In the presence of adenosine, it selectively binds to the aptamer, displacing the catalytic chain into the solution. The cyclic portion of H1 hybridizes with the catalytic strand, while H2 hybridizes with the exposed H1 fragment to form an H1/H2 complex containing a G-quadruplex. Thioflavin T binds specifically to the G-quadruplex, generating a fluorescent signal. As a nucleic acid chaperone, PLL-*g*-Dex expedites the strand exchange reaction, enhancing the efficiency of catalytic hairpin assembly, thus amplifying the signal and reducing detection time. The optimal detection conditions were determined to be a temperature of 25 °C and a reaction time of 10 min. Demonstrating remarkable sensitivity and selectivity, the sensor achieved a lowest limit of detection of 9.82 nM. Furthermore, it exhibited resilience to interference in complex environments such as serum, presenting an effective approach for rapid and sensitive adenosine detection.

## 1. Introduction

Adenosine is a ubiquitous nucleoside substance that plays a crucial role in various physiological processes such as neurotransmission, immune response regulation, and cardiovascular function regulation [1,2,3]. It functions not only as a neuromodulator and vasodilator, influencing physiological processes such as sleep regulation, heart function, and blood flow, but also participates in inflammatory responses and immune system activity regulation [4,5,6]. Numerous studies have demonstrated a significant correlation between adenosine expression levels and the onset and progression of cancer. Consequently, adenosine is considered a potential diagnostic marker for cancer [7]. For instance, in lung cancer cases, plasma adenosine levels are markedly higher compared to those of healthy individuals. However, despite the variety of reported adenosine detection methods, including colorimetric analysis [8], surface-enhanced Raman spectroscopy [9], high-performance liquid chromatography [10], and electrochemical methods [11], each method has its limitations. For instance, some methods may suffer from inadequate sensitivity or involve cumbersome sample preparation. Specifically, while high-performance liquid chromatography offers high sensitivity, its selectivity can constrain its applicability in certain scenarios. Therefore, it is particularly urgent to develop a rapid and sensitive adenosine detection approach.

Recently, fluorescent biosensors have garnered significant attention due to their advantages, including high sensitivity, real-time monitoring capabilities, and rapid response to biomolecules [12,13]. To further enhance the performance of these biosensors, researchers typically integrate them with various signal amplification strategies, such as hybridization chain reaction (HCR) [14], catalytic hairpin assembly (CHA) [15], and polymerase chain reaction (PCR) [16]. CHA utilizes the unique properties of the hairpin structure to initiate a series of assembly reactions in the presence of target molecules, achieving signal amplification, albeit with stricter reaction conditions. The cationic comb-type copolymer poly(L-lysine)-graft-dextran (PLL-*g*-Dex) effectively promotes DNA hybridization, accelerates strand displacement reactions, and stabilizes the multi-level structure of DNA at room temperature, which is defined as chaperone activity [17,18,19]. Prof. Maruyama’s lab has demonstrated that PLL-*g*-Dex enhances the reaction rate of both HCR and CHA [20,21]. Consequently, PLL-*g*-Dex is employed to enhance the sensitivity of sensors and reduce the detection time, and some chaperone copolymers-based sensors have been successfully developed to highly detect nucleic acids and proteins [22,23].

The G-quadruplex is a complex secondary structure formed by sequences rich in guanine in DNA or RNA that fold in a specific manner. Thioflavin T (ThT) is a small-molecule fluorescent dye that exhibits strong fluorescence upon specific binding to G-quadruplexes. This property renders it widely applicable across numerous fields [24,25]. For example, in biosensor development, Liu et al. devised a sensor utilizing rolling circle amplification in conjunction with G-quadruplexes to detect apurinic/apyrimidinic endonuclease (APE1). The fluorescence signal is generated through the interaction of G-quadruplexes with ThT [26]. Furthermore, fluorescent biosensors constructed based on the binding mechanisms of G-quadruplexes and thioflavin T find utility in the detection of metal ions, nucleic acids, and proteins [27,28,29]. Compared to conventional methods reliant on labeling, this label-free approach not only simplifies experimental procedures and reduces the detection cycle but also diminishes overall experimental costs.

In this study, we developed a DNA biosensor utilizing a streptavidin magnetic beads probe and a cationic comb-type copolymer to facilitate catalytic hairpin assembly for the sensitive detection of adenosine. Initially, a functional probe consisting of streptavidin magnetic beads, aptamer, and catalytic chain was synthesized. In the presence of adenosine, it preferentially binds to the aptamer, displacing the catalytic chain into the solution. Subsequently, the cyclic part of H1 hybridizes with the catalytic strand, leading to the opening of H1, followed by H2 hybridizing with the exposed H1 fragment to form an H1/H2 complex with a G-quadruplex. The addition of PLL-*g*-Dex promotes chain exchange, thereby enhancing the reaction efficiency of catalytic hairpin assembly. ThT interacts with more G-quadruplexes to amplify the signal. The sensor exhibits a low limit of detection and offers a rapid and sensitive approach for quantifying adenosine.

## 2. Materials and Methods

### 2.1. Materials

The DNA utilized in this experiment (Table 1) was sourced from Sangon Bioengineering Co., Ltd. (Shanghai, China) and purified via HPLC. Adenosine (AD) and poly-L-lysine were procured from Sigma-Aldrich Trading Co., Ltd. (Shanghai, China). Thioflavin T (ThT) was acquired from Beijing Solebao Technology Co., Ltd. (Beijing, China). Potassium chloride (KCl) and sodium chloride (NaCl) were obtained from Aladdin Co., Ltd. (Shanghai, China). Ultrapure water was purchased from Guangzhou Dongsheng Biotechnology Co., Ltd. (Guangzhou, China). Tween-20 was obtained from Jiangsu Kaiji Biotechnology Co., Ltd. (Suzhou, China). The pH reference solution was sourced from Shanghai Myrrell Biochemical Technology Co., Ltd. (Shanghai, China). Streptavidin magnetic beads (MBs, 50 mg/mL, diameter 500 nm), Tris-HCl buffer (1 mol/L, pH = 7.4), and TE buffer were procured from Sangon Bioengineering Co., Ltd. (Shanghai, China). Cytidine, guanosine, and uridine were acquired from Shanghai Yuanye Biotechnology Co., Ltd. (Shanghai, China).

### 2.2. Experimental Equipment

Fluorescence signals were assessed employing an RF-6000 fluorescence spectrometer (Shimadzu, Kyoto, Japan). ThT served as the fluorescent dye in this experiment, exhibiting specific binding to the G-quadruplex structure, thus generating a fluorescent signal. The fluorescence detection employed an excitation wavelength of 420 nm and an emission wavelength of 460 nm. Spectral scanning ranged from 460 nm to 580 nm, with excitation and emission light bandwidths of 5 nm and 10 nm, respectively.

### 2.3. Preparation of Reagents

The DNA was diluted to 100 µM with ultrapure water and stored. The TTL solution was prepared from 0.1% Tween-20, 100 mM Tris, and 1 M LiCl reference solution. The ThT was dissolved in ultrapure water and diluted to a concentration of 50 µM. The TN buffer consisted of 50 mM Tris and 100 mM NaCl. The KCl was dissolved in Tris-HCl buffer (50 mM Tris). All solutions were stored at 4 °C until needed. H1 and H2 were heated at 95 °C for 5 min, then slowly cooled to room temperature overnight to ensure stable formation of the hairpin structure.

### 2.4. Synthesis of PLL-g-Dex

PLL-*g*-Dex was synthesized by a reductive amination reaction, as mentioned previously [23]. The dextran content of PLL-*g*-Dex was 91 wt%, determined by ^1^H-NMR (Figure S1). The infrared spectra of PLL, Dextran, and PLL-*g*-Dex are shown in Figure S2. The infrared and ^1^H-NMR spectra proved that the structure of the copolymer was consistent with what was expected.

### 2.5. Preparation of Streptavidin Magnetic Beads Probe

First, 50 µL of streptavidin magnetic beads were separated using a magnetic stand, and the storage solution was removed. The streptavidin magnetic beads underwent three washes with 1 mL of TTL buffer to ensure complete removal of non-specific binding substances from their surface. Subsequently, 50 µL of aptamer (10 µM) and 100 µL of tDNA (6.5 µM) were individually heated at 95 °C for 5 min and then quickly moved and held at −20 °C for 10 min to prevent dimer formation. Afterward, 50 µL of aptamer was added to the washed magnetic beads and continuously shaken on a vortex oscillator for 30 min. Following magnetic separation, the supernatant was discarded, 100 µL of tDNA was added, and it was incubated at 37 °C for 1 h. The beads were washed twice with TN buffer. Finally, the prepared magnetic beads probes were evenly distributed into 200 µL of TN buffer and stored at 4 °C until use.

### 2.6. Fluorescence Detection

25 µL of streptavidin magnetic beads probe was combined with 25 µL of adenosine solution and incubated at 37 °C for 1 h [30]. After magnetic separation, 5 μL each of H1, H2, K^+^, ThT, and PLL-*g*-Dex were added to the supernatant and diluted to 200 μL with Tris-HCl buffer. This mixture was then incubated at 25 °C for 10 min. Finally, the prepared solution was transferred to a cuvette for fluorescence detection.

## 3. Results and Discussion

### 3.1. Principle of Adenosine Detection

**Figure 1:**
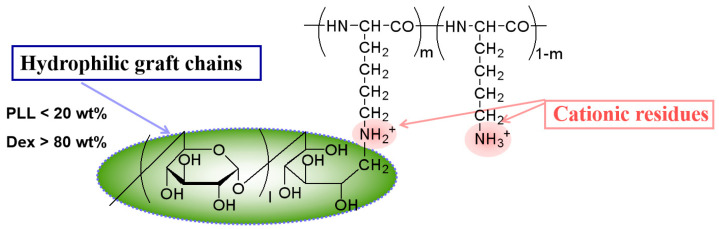
Chemical structure of chaperone copolymer PLL-*g*-Dex.

In our previous work, we demonstrated that the chaperone copolymer PLL-*g*-Dex (Figure 1), consisting of a polycationic backbone and more than 80 wt% hydrophilic graft chains, as a model for artificial nucleic acid chaperone, significantly accelerates DNA hybridization and strand-exchange reactions. Additionally, compared with those in buffer only, the stability of DNA multi-level structures is also increased in the presence of PLL-*g*-Dex [31,32,33] The mechanism is that chaperone copolymers reduce the entropy-adverse anti-ionic condensation effect during DNA hybridization, and reduce the energy barrier associated with the break and recombination of nucleic acid base pairs. Thus, PLL-*g*-Dex would be suggested to act as a nucleic acid chaperone, activating appropriate hybridization and strand-exchange reactions, which is useful for sensitive DNA analyses, and especially for signal amplification during hybridization processes.

**Figure 2:**
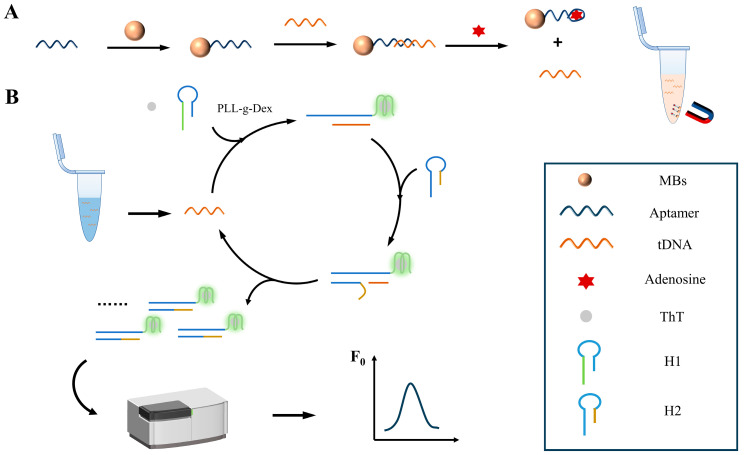
Schematic diagram of a biosensor based on (**A**) streptavidin magnetic beads functional probe, and (**B**) PLL-*g*-Dex assisted catalytic hairpin assembly for adenosine detection.

Figure 2 illustrates the operational mechanism of a biosensor utilizing a streptavidin magnetic beads functional probe and a PLL-*g*-Dex-assisted catalytic hairpin assembly for adenosine detection. Initially, we devised a functional magnetic beads probe comprising streptavidin magnetic beads, tDNA, and an aptamer. Through the specific binding of the biotin-containing aptamer to streptavidin, the aptamer becomes immobilized on the magnetic beads surface. Subsequently, tDNA is introduced and hybridizes with the aptamer due to sequence complementarity, forming the magnetic beads probe. In the presence of adenosine, the aptamer preferentially binds to it, leading to the displacement of tDNA. Under an external magnetic field, the magnetic beads partially separate, and the displaced tDNA acts as a catalytic strand, initiating the catalytic hairpin assembly process, as shown in Figure 2A.

The chaperone copolymer-assisted catalytic hairpin assembly program for adenosine detection is shown in Figure 2B. We designed two hairpin DNAs, H1 and H2, where the G-quadruplex sequence is located in the green region of H1. The catalytic chain tDNA pairs with the loop sequence of hairpin structure H1, opening it to form a tDNA/H1 complex. In the presence of K^+^, guanine-rich sequences form G-quadruplex structures. Subsequently, the base sequence of the toe part of H2 complements the exposed blue area of the tDNA/H1 complex, displacing tDNA. This cyclic process continuously recycles tDNA as a catalytic chain, generating additional G-quadruplex structures. Introducing PLL-*g*-Dex facilitates the chain exchange process and stabilizes the H1/H2 complex with a G-quadruplex structure, thereby enhancing the efficiency of CHA and shortening the detection time. The specific binding of ThT to G-quadruplex structures results in the generation of fluorescent signals. In the absence of adenosine in the system, the streptavidin magnetic beads probe carrying tDNA is isolated through the application of an external magnetic field. Only the two hairpin structures, H1 and H2, persist in the solution, and remain relatively stable. Thus, no fluorescence signal is generated.

### 3.2. Feasibility Analysis

In this experiment, the presence of target adenosine replaced the tDNA on the streptavidin magnetic beads probe, acting as the initiator strand to facilitate subsequent catalytic hairpin assembly, resulting in the formation of a G-quadruplex structure. The addition of ThT produced a fluorescence signal, with varying concentrations of adenosine leading to different fluorescence intensities. Higher concentrations of target adenosine displaced more tDNA, increasing the likelihood of hairpin DNA opening and G-quadruplex structure formation, thereby elevating the fluorescence value.

**Figure 3:**
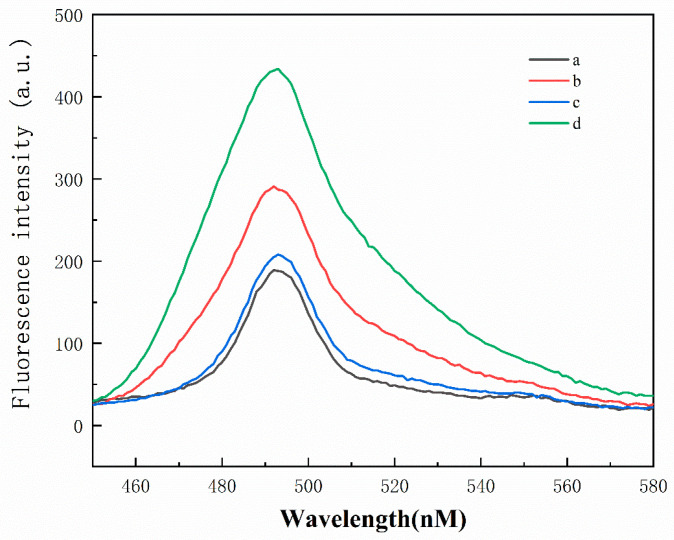
Fluorescence emission spectrum under different conditions: (a) MBs-Aptamer/tDNA + H1 + H2 + K^+^; (b) MBs-Aptamer/tDNA + H1 + H2 + K^+^ + Target; (c) MBs-Aptamer/tDNA + H1 + H2 + K^+^ + PLL-*g*-Dex; and (d) MBs-Aptamer/tDNA + H1 + H2 + K^+^+ PLL-*g*-Dex + Target.

The sensor’s feasibility was confirmed by assessing the fluorescence signal intensity under varying conditions. Figure 3 shows the fluorescence emission spectra recorded with different components. In the presence of adenosine, it competes with tDNA for binding to the aptamer on the streptavidin magnetic beads probe. Due to the aptamer’s specific recognition of adenosine, adenosine predominantly occupies the aptamer, freeing tDNA into the solution. In the presence of PLL-*g*-Dex, tDNA serves as a catalytic chain, facilitating the opening of hairpin H1. The exposed portion of H1 then hybridizes with the loop region of H2. The displaced tDNA perpetuates this cycle, leading to the formation of additional DNA complexes with G-quadruplexes, resulting in a pronounced fluorescence signal in conjunction with ThT (curve d). This enhancement is attributed to PLL-*g*-Dex expediting the chain exchange reaction, thereby enhancing the efficiency of CHA. Conversely, the absence of PLL-*g*-Dex leads to decreased fluorescence signal intensity (curve b). In the absence of adenosine, the streptavidin magnetic beads probe remains unaffected, and tDNA continues to hybridize with the aptamer, failing to trigger subsequent reactions and resulting in only a faint background signal (curve a). Curve c demonstrates the scenario where adenosine is absent, but PLL-*g*-Dex is present, yielding a slightly higher fluorescence intensity than the background signal. These findings indicate that PLL-*g*-Dex exerts negligible influence on the control group lacking the target substance. In conclusion, aside from the entire system, the fluorescence intensity of the other components remains low, thus validating the feasibility of this strategy for adenosine detection.

### 3.3. Optimization of Experimental Conditions

**Figure 4:**
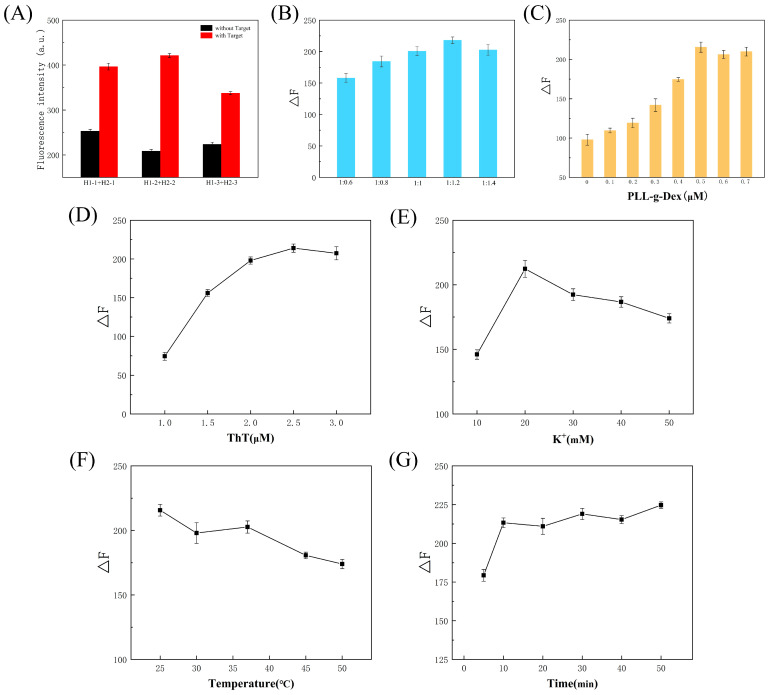
Optimization of experimental conditions. (**A**) Sequence of H1 and H2. (**B**) Concentration of H1 and H2. (**C**) Concentration of PLL-*g*-Dex. (**D**) ThT concentration. (**E**) K^+^ concentration. (**F**) Reaction temperature. (**G**) Reaction time.

To enhance the sensor’s detection efficiency, we optimized various experimental conditions. The proper design of DNA sequences significantly impacts the success of sensors. To ensure precision, we designed three groups of hairpin DNA in this experiment: H1-1 + H2-1, H1-2 + H2-2, and H1-3 + H2-3. The experimental results are depicted in Figure 4A. In the presence of adenosine, the fluorescence intensity initially increased and then decreased as the bases of the DNA stem grew. Conversely, in the absence of adenosine, the background signal was higher due to the shorter DNA stem, which facilitated the formation of a G-chain structure. Increasing the number of bases in the stem enhanced the stability of the hairpin DNA. Optimal signal acquisition was achieved by selecting H1-2 and H2-2 as the most effective sequences.

The concentration of DNA used significantly influences the accuracy and reliability of sensor experiments and consequently affects the reaction efficiency. H1 and H2 jointly constituted a crucial aspect of the catalytic hairpin assembly in this study. Their concentrations directly impacted the efficiency of this process. Figure 4B illustrates the experimental outcomes using varying concentration ratios of H1 and H2. H1 was consistently set at 200 nM. With increasing H2 concentration, fluorescence intensity gradually rose, peaking at a concentration ratio of H1 to H2 of 1:1.2. Subsequent increments in H2 concentration yielded marginal changes in fluorescence intensity. This indicates that while elevated H2 concentrations promoted catalytic hairpin assembly, they also resulted in heightened background signals. Consequently, the final chosen concentrations for H1 and H2 were 200 nM and 240 nM, respectively.

The concentration of PLL-*g*-Dex significantly impacts the efficiency of catalytic hairpin assembly in experiments such as ours. Adding an optimal quantity of PLL-*g*-Dex expedited the strand exchange reaction, leading to the formation of more target DNA complexes with G-quadruplexes in less time. This facilitated the specific binding of ThT to additional G-quadruplex structures, thereby enhancing fluorescence intensity. As depicted in Figure 4C, the fluorescence intensity initially increased with rising PLL-*g*-Dex concentrations before stabilizing. It peaked at a concentration of 0.5 µM. Subsequently, further increases in concentration yielded minimal changes in fluorescence intensity, or even slight decreases. This phenomenon may have arisen because excessive PLL-*g*-Dex stabilized the DNA chain, hindering the strand displacement reaction and elevating background signals. Conversely, an optimal PLL-*g*-Dex concentration promoted the strand displacement reaction, augmenting the formation of target DNA complexes. Therefore, we identified 0.5 µM as the optimal concentration of PLL-*g*-Dex.

The concentration of ThT significantly influences sensor design, as it acts as a fluorescent dye that binds specifically to G-quadruplexes. Figure 4D illustrates that as ThT concentration increased, so did fluorescence intensity, peaking at 2.5 µM. The fluorescence intensity experienced a notable increase when the ThT concentration ranged from 1.0 µM to 2.0 µM. However, within the concentration range of 2.0 µM to 3.0 µM, the fluorescence intensity exhibited minimal variation, maintaining a relatively stable trend. Increased ThT binding to the G-quadruplex led to enhanced fluorescence intensity. However, once the ThT concentration surpassed a certain threshold, the fluorescence signal became saturated, resulting in minimal changes in fluorescence intensity, even with additional ThT. Consequently, 2.5 µM was deemed the optimal ThT concentration.

The presence of K^+^ ions facilitates the formation of a stable G-quadruplex structure in single-stranded DNA rich in guanine sequences. As illustrated in Figure 4E, the fluorescence intensity increased significantly from 10 mM to 20 mM of K^+^ concentration, reaching its peak. However, further increases in K^+^ concentration led to a decline in fluorescence intensity. Even slight variations in K^+^ concentration resulted in changes in fluorescence intensity. Therefore, maintaining an appropriate K^+^ concentration is crucial for ensuring the optimal stability of the G-quadruplex structure. Hence, we selected 20 mM as the optimal K^+^ concentration.

Temperature fluctuations can significantly impact the hybridization efficiency between DNA molecules, thus influencing sensor performance. Typically, elevated temperatures accelerate DNA reaction rates, yet they can also weaken hydrogen bonds within the stem of hairpin DNA, leading to easier dissociation and decreased hairpin DNA stability, even in the absence of the target. Conversely, higher temperatures yield heightened fluorescence signal values. Figure 4F illustrates experimental optimizations at various temperatures, revealing peak fluorescence intensity at 25 °C. Subsequent temperature increases correlate with a decline in fluorescence intensity disparity. This trend may stem from reduced DNA stability and heightened background signals at elevated temperatures. Consequently, 25 °C was selected as the optimal reaction temperature based on experimental findings.

The duration of the reaction time significantly influences a sensor’s detection performance. A too-brief reaction time results in inadequate DNA hybridization, diminishing sensor accuracy, while an excessively long duration increases experimental costs and causes unnecessary losses. Figure 4G illustrates that in the present experiment, prolonged reaction times initially boosted fluorescence intensity before stabilizing. The change in fluorescence intensity became less pronounced as the reaction time extended from 10 to 50 min. Insufficient time hampered hairpin H1 opening, reducing the formation of DNA complexes with G-quadruplex structures and weakening the fluorescence signal. Conversely, extending the reaction time did not yield additional fluorescence signals. Economically, shortening the reaction time reduced unnecessary experiment losses and enhances sensor detection efficiency. Therefore, 10 min was selected as the optimal reaction time.

### 3.4. Detection Performance of Sensor

The sensor was designed to achieve rapid, enzyme-free, and highly sensitive detection of adenosine. We utilized the optimal experimental conditions derived from previous experiments to assess the sensor’s detection performance. Specifically, we selected the final DNA sequences of H1-2 and H2-2, maintaining a concentration ratio of H1 to H2 at 1:1.2. Concurrently, PLL-*g*-Dex was set at 0.5 µM, ThT at 2.5 µM, and K^+^ at 20 mM, with a reaction temperature of 25 °C and a reaction time of 10 min. Under these conditions, the fluorescence signal reached its maximum value. Subsequently, we evaluated various concentrations of adenosine under these optimized conditions to establish the relationship between adenosine concentrations and fluorescence intensity.

**Figure 5:**
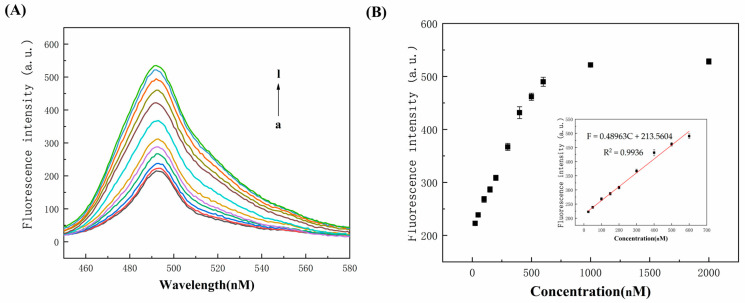
(**A**) Fluorescence spectra of different adenosine concentrations (a–l: 0, 25, 50, 100, 150, 200, 300, 400, 500, 600, 1000, and 2000 nM). (**B**) The correlation between adenosine concentrations and fluorescence signal intensity (the inset shows the linear calibration curve of adenosine vs. fluorescence intensity in the range of 0–600 nM). The error bars represent standard deviations obtained from triplicate experiments.

As shown in Figure 5A, the fluorescence signal increased as the adenosine concentration in the system rose from 25 nM to 2000 nM. This indicates that higher adenosine levels can displace tDNA bound to the streptavidin magnetic beads probe, enabling it to act as the initiating strand in subsequent steps, thus facilitating the formation of DNA complexes and the production of more G-quadruplex structures, thereby amplifying the signal. Figure 5B illustrates a plot of fluorescence intensity against adenosine concentration. Following linear regression, a strong linear relationship was observed within the adenosine concentration range of 25 nM to 600 nM, yielding the regression equation F = 0.48963C + 213.5604 (where C represents the concentration of adenosine) with an R^2^ value of 0.9936 and a detection limit of 9.82 nM. The detection limit was calculated using 3 σ/S, where σ denotes the standard deviation of the blank signal. Compared with other methods (Table 2), the sensing strategy based on cationic copolymer-assisted catalytic hairpin assembly demonstrated a lower detection limit, indicating its potential for detecting adenosine concentration.

### 3.5. Specificity Analysis

**Figure 6:**
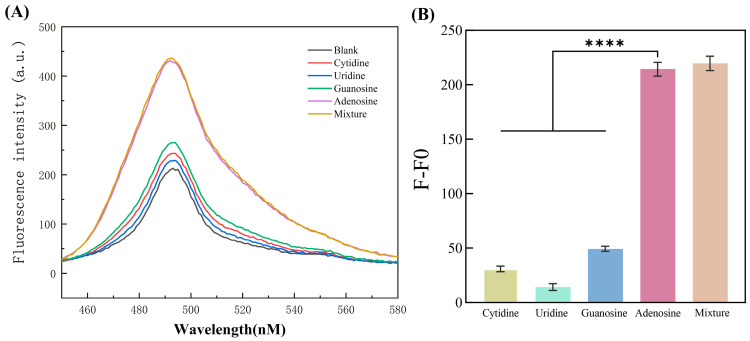
(**A**) Fluorescence spectrum diagram of the relationship between different types of nucleosides and fluorescence intensity. (**B**) Diagram of the relationship between the fluorescence intensity difference and different nucleoside types at the excitation wavelength of 420 nm. Statistical significance was calculated by the one-way ANOVA. (**** *p* < 0.0001).

The sensor’s resistance to interference from other substances is a crucial indicator that warrants attention. In the assessment of sensor performance, selectivity emerges as a pivotal criterion. A sensor with high specificity solely reacts to its designated target, thereby yielding precise and dependable measurements. To investigate the sensor’s selectivity for the target molecule adenosine, we conducted five sets of experiments aimed at detecting various nucleosides within the same family, including cytidine, guanosine, uridine, adenosine, and mixed nucleosides. The results, illustrated in Figure 6, indicated that the fluorescence intensity of the system containing adenosine far surpassed that of other nucleosides individually. In the experiments, the concentration of adenosine was maintained at 400 nM, and even with the addition of other nucleosides, the fluorescence intensity remained largely unchanged. This observation suggests that the aptamer on the streptavidin magnetic beads probe exhibited a stronger affinity for adenosine. Compared to the presence of other nucleosides, adenosine demonstrated greater competitiveness as a tDNA competitor, thereby facilitating the release of more tDNA. As the pivotal initiator strand of the catalytic hairpin assembly, tDNA enhanced the efficiency of DNA complex formation, consequently generating more G-quadruplex structures to amplify fluorescent signals. In conclusion, the experimental results demonstrated that the sensor employing cationic comb-type copolymer-assisted catalytic hairpin assembly exhibited favorable selectivity.

### 3.6. Detection in Serum Samples

The ability to detect compounds in complex matrices like serum is a fundamental and pivotal aspect of sensor performance assessment. To ascertain the applicability of our method in detecting adenosine within serum samples, we introduced three distinct concentrations of adenosine (100 nM, 200 nM, and 500 nM) into diluted serum specimens for recovery experiments. The experimental findings are presented in Table 3, indicating recovery rates ranging from 95.52% to 99.57% and relative standard deviations ranging from 0.46% to 2.34% for the recovery rate. The recovery rate experiment served as a critical metric for assessing the sensor’s capability to precisely identify and quantify the target analyte. Notably, the results indicate high accuracy and a notable resilience to interference, underscoring the sensor’s efficacy in serum-based adenosine detection.

In order to evaluate the biocompatibility of chaperone copolymers, the survival and proliferation of RAW 264.7 cells cultured with PLL-*g*-Dex in vitro were studied. With an increase in culture time, RAW 264.7 cells gradually proliferated in plates with or without PLL-*g*-Dex (control group), as shown in Figure S3A. The proliferation rate of RAW 264.7 cells supplemented with PLL-*g*-Dex was higher than that of cells without PLL-*g*-Dex. A Live/Dead Assay Kit was used to test cell viability. All samples showed high cell viability (green), as shown in Figure S3B. Interestingly, more cells were observed on PLL-*g*-Dex, showing good cell viability and proliferation. Therefore, PLL-*g*-Dex has good cytocompatibility, and thus possesses potential application value in the biotechnology field.

## 4. Conclusions

This paper presents an enzyme-free and label-free strategy for highly sensitive detection of adenosine, utilizing cationic comb-type copolymer-assisted CHA. Initially, the sensor constructs a streptavidin magnetic beads probe to isolate and separate adenosine, shifting the focus from adenosine concentration detection to targeting the chain. Serving as the priming strand of CHA, the target strand hybridizes with the cyclic base sequence of hairpin H1. Subsequently, hairpin H2 binds with the opened H1, displacing the target strand and acting as the priming strand for the next CHA cycle. The involvement of PLL-*g*-Dex promotes the strand exchange reaction, thereby enhancing the reaction efficiency of CHA. ThT is integrated into the G-quadruplex structure to generate strong fluorescence. The designed fluorescence sensor effectively detects adenosine molecules with a detection limit of 9.82 nM and a linear range spanning from 25 nM to 600 nM. Additionally, the good recovery rate in serum samples indicates the sensor’s anti-interference capability. Due to the good performance of this sensor, it has certain application prospects in detecting adenosine in the biomedical field.

## Figures and Tables

**Table 1:** Dry base ratio of rubber auxiliaries in vulcanization mixture.

Name	Sequence (5′-3′)
Aptamer	biotin-TTTTTTTTTACCTGGGGGAGTATTGCGGAGGAAGGT
tDNA	TTCCTCCGCAATGATAGATA
H1-1	CCACCCATTCCTATGTATCTATCATTGCGGAGGAATGGGTGGGTGGGTGGG
H2-1	ATTCCTCTGTATCTATTCCTCCGCAATGATAGATACATAGGAATGGGTGG
H1-2	CCACCCAATTCCTCATATCTATCATTGCGGAGGAATTGGGTGGGTGGGTGGG
H2-2	AATTCCTCATATCTATTCCTCCGCAATGATAGATATGAGGAATTGGGTGG
H1-3	CCACCCACATTCCTCCATATCTATCATTGCGGAGGAATGTGGGTGGGTGGGTGGG
H2-3	ACATTCCTCCATATCTGATTCCTCCGCAATGATAGATATGGAGGAATGTGGGTGG

**Table 2:** Comparison of different methods for detecting adenosine.

Detection Technique	Linear Range	LOD	Reference
Electrochemistry	0.37–37.4 µM	210 nM	[34]
Electrochemistry	5–75 nM	3.1 nM	[35]
Colorimetry	60–280 nM	21 nM	[36]
Fluorescence	0.5–20 µM	84 nM	[37]
Fluorescence	25–600 nM	9.82 nM	This work

**Table 3:** Detection results of adenosine in serum samples.

Added (nM)	Found (nM)	Recovery (%)	RSD (%, *n* = 3)
100	95.52	95.52%	2.34
200	193.67	96.84%	1.66
500	497.87	99.57%	0.46

## Data Availability

The raw data supporting the conclusions of this article will be made available by the authors on request.

## Supplementary Materials

The following supporting information can be downloaded at: https://www.mdpi.com/article/10.3390/polym16152179/s1, Figure S1: (A) Structural formula of poly(L-lysine)-graft-dextran (PLL-*g*-Dex) copolymer. (B) ^1^H-NMR spectra in D_2_O (400 MHz) of PLL, Dextran, and PLL-*g*-Dex. Figure S2: The infrared spectra of PLL, Dextran and PLL-*g*-Dex. Figure S3: (A) Proliferation of the RAW 264.7 cells cultured on the surfaces of the PLL-*g*-Dex or the culture plates (without PLL-*g*-Dex). (B) Viability and morphology of the RAW 264.7 cells cultured on surfaces of (a) the culture plates and (b) PLL-*g*-Dex for 24 h. Table S1: The *t*-test for adenosine in serum samples.
