# Supplementary material for: Chaperone Copolymer-Assisted Catalytic Hairpin Assembly for Highly Sensitive Detection of Adenosine
Source: Polymers (Basel). 2024 Jul 31;16(15):2179. doi: 10.3390/polym16152179 (PMC11314456; doi:10.3390/polym16152179)
Supplement: Supplementary file 1 [file polymers-16-02179-s001.zip › polymers-3099509-supplementary.pdf]

Supporting Information

Chaperone Copolymer-assisted Catalytic Hairpin Assembly for Highly Sensitive Detection of Adenosine

Yazhen Liao †, Xiaoxue Yin †, Wenqian Liu, Zhenrui Du, Jie Du*

School of Materials Science and Engineering, Hainan University, Haikou 570228, PR China.

*Corresponding author: dujie@hainanu.edu.cn

† Y. Liao and X. Yin contributed equally to this paper

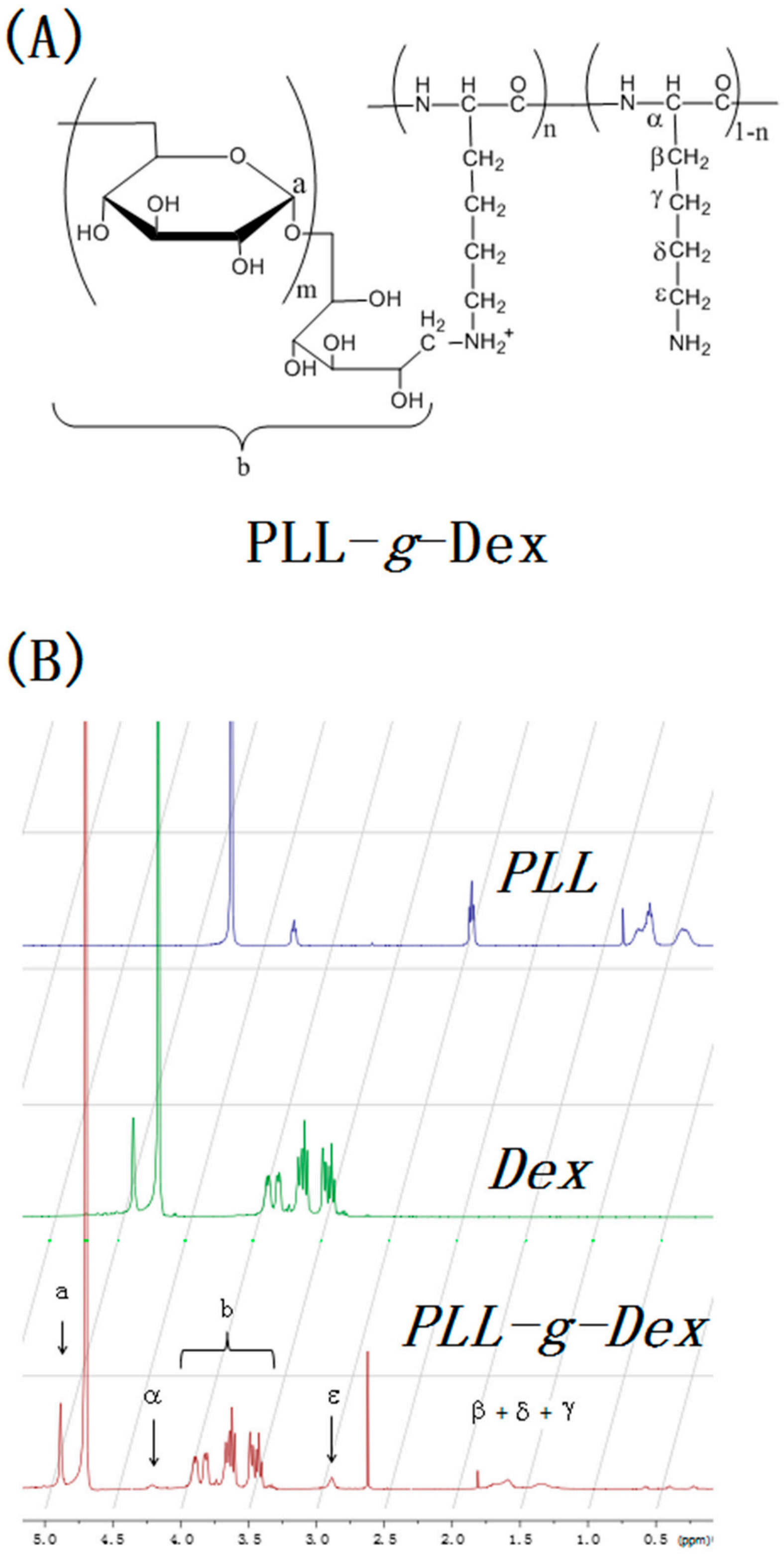

Figure S1 (A) Structural formula of poly(L-lysine)-graft-dextran (PLL-*g*-Dex) copolymer. (B) ^{1}H-NMR spectra in D_2O (400 MHz) of PLL, Dextran, and PLL-*g*-Dex. The dextran content of the copolymer was calculated from ^{1}H-NMR signals assigned to PLL (ε -CH_2) and dextran (C_1-H, a).

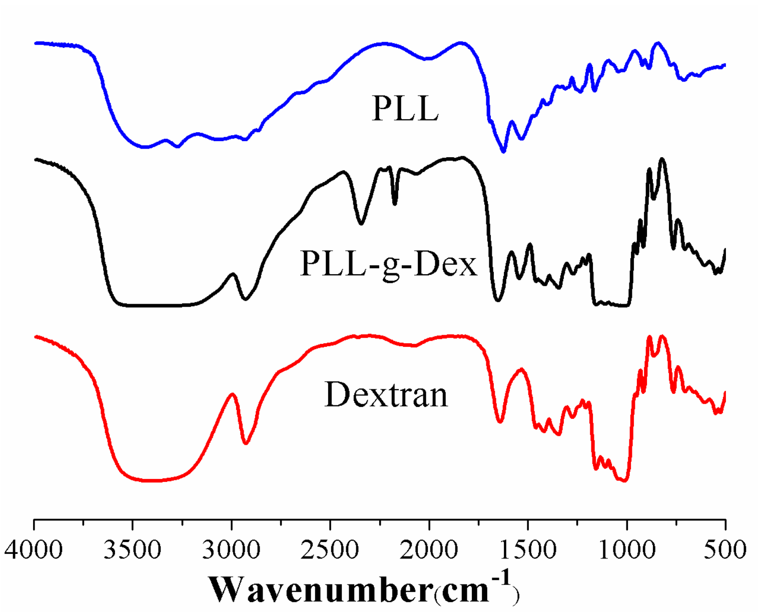

Figure S2 The infrared spectra of PLL, Dextran and PLL-*g*-Dex

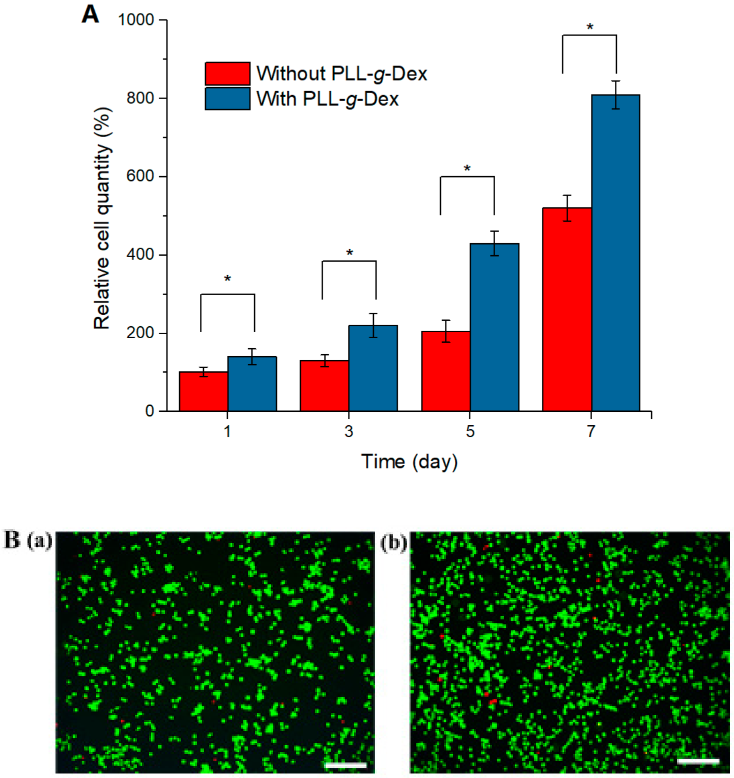

Figure S3 (**A**) Proliferation of the RAW 264.7 cells cultured on the surfaces of the PLL-*g*-Dex or the culture plates (without PLL-*g*-Dex). * p <0.05. (**B**) Viability and morphology of the RAW 264.7 cells cultured on surfaces of (a) the culture plates and (b) PLL-*g*-Dex for 24 h (green cells are live, and red cells are dead; the scale bar is 200 µm).

Ethics Approval Statement. The human research in this study were prospectively reviewed and approved by the ethics committee (Ethics Approval 2022-(ER)−047) of Haikou People's Hospital, China. The human research were performed on subjects who provided informed consent before participating.

Table S1 The t-test for adenosine in serum samples.

Added（nM）	Found（nM）	Recovery（%）	t	p
	97.01	97.01		
100.00	96.61	96.61	-3.465	0.148
	92.95	92.95		
	190.84	95.42		
200.00	193.03	96.52	-3.413	0.152
	197.16	98.58		
	499.95	99.99		
500.00	495.38	99.08	-1.599	0.502
	498.27	99.65		

Statistical Analysis. The software GraphPad GraphPad Prism 10 was used for statistical analyses. ach experiment was performed in triplicate, and the one-way ANOVA (and non-parametric) statistical test was used to evaluate the data. The P value < 0.05 was deemed to be statistically significant.
